# Hereditary Hyperferritinemia-Cataract Syndrome in 3 Generations of a Family in East Tennessee

**DOI:** 10.1155/2020/2837573

**Published:** 2020-05-26

**Authors:** Heidi A. Worth, Zachary Marlette, David Aljadir, Ronald Lands

**Affiliations:** Department of Medicine, Division of Hematology/Oncology, University of Tennessee Medical Center, Knoxville, TN, USA

## Abstract

Hereditary hyperferritinemia and cataracts syndrome (HHCS) without iron overload is a syndrome first identified less than 3 decades ago. While investigators have dissected the gene where several responsible mutations reside, it remains a relatively unknown genetic disorder to clinicians. The result is often an expensive, invasive evaluation for iron overload, followed by a well-intended prescription for a series of phlebotomies that delivers morbidity instead of benefit. We present a father with an elevated ferritin and heterozygosity for H63D HFE mutation whose clinical course followed this path. His treatment rendered him symptomatic from iron deficiency with no reduction in his ferritin. On re-evaluation, a review of his past medical history clarified the cataract surgery noted in his record had occurred at a young age. Furthermore, one of his daughters required cataract surgery as a teenager. With this information, we strongly suspected HHCS. His phlebotomies were discontinued, and within weeks, his iatrogenic iron deficiency resolved and his health returned to normal.

## 1. Introduction

Ferritin is a protein that binds and sequesters intracellular iron. It is a hollow sphere where iron is oxidized and stored, composed of 24 heavy and light subunits in variable proportions in different tissues [1]. It is an indicator of iron stores. When low, it indicates iron deficiency. When elevated, it may indicate iron overload as in HFE hemochromatosis, thalassemia, and multiple blood transfusions. More commonly, it is elevated with very different implications due to a broad range of inflammatory states not associated with iron overloading including metabolic syndrome, autoimmune disease, chronic and acute infections, malignancy, and release of ferritin from hepatocytes in liver disease.

Hereditary hyperferritinemia-cataract syndrome (HHCS) was first described in 1995 in a family suspected of having a new genetic disease involving the contiguous L-ferritin gene (FTL) and the gene coding for the lens membrane protein thus accounting for the linkage of hereditary cataracts and elevations of L-ferritin [2]. Separate investigators soon reported another family with mutations in the iron-responsive element (IRE) of the L-ferritin gene that removed the inhibitory interaction with iron regulator protein (IRP) and deregulation of ferritin synthesis [3]. Since the initial discovery, other families in Western Europe, Australia, India, and the United States have been described. Over 32 different mutations were reported by 2013 [4]. The IRE, which is a noncoding stem loop, located in the 5′ untranslated region (5′ UTR) of L-ferritin mRNA plays an important role in the regulation of ferritin synthesis through its interaction with the IRP. Typically, the synthesis of ferritin is regulated by iron availability. When sufficient iron is present, a change in structure of the IRE occurs, preventing its binding to the IRP, leading to ferritin synthesis. In contrast, when levels of cellular iron are low, no change in structure of the IRE occurs, thus allowing the binding of IRP with IRE and inhibiting the translation of ferritin. The mutations of the IRE associated with HHCS lead to a structural change inhibiting the binding with the IRP, allowing for deregulated production of L-ferritin irrespective of cellular iron stores. The increased L-ferritin is due to increased synthesis and not due to elevated iron stores, so the serum iron and transferrin saturation remain normal. There are reports of associations of specific mutations in the IRE with the serum ferritin level, and the age of onset and severity of the cataracts. Other genetic and environmental modifiers are likely to play a role [5].

## 2. Case Presentation

A 61-year-old Caucasian male was referred to a hematologist for a presumed diagnosis of hemochromatosis after laboratory ordered in the course of evaluation of elevated transaminases demonstrated an elevated ferritin of 1,372 ug/L, and genetic testing demonstrated heterozygosity for H63D mutation. He was active and healthy, exercised regularly, and was a recreational runner and hiker. His past medical history was remarkable for bilateral cataract removal in separate procedures at the age of 12 years and at age 28. Family history was significant in that his father also had cataract surgery in separate procedures at age 40 and 60 years. One of the patient's daughters required surgery for congenital cataracts at the age of 15.

He was initially thought to have iron storage disease based on the presence of the heterozygous H63D mutation and the elevated ferritin. He was prescribed a series of monthly phlebotomies with no measurable reduction in his ferritin levels. A more aggressive treatment with weekly phlebotomies was abandoned when he developed a profound fatigue syndrome, exercise intolerance, and a microcytic anemia. Despite being symptomatic with iron deficiency, his serum ferritin remained elevated at levels consistently >1000 mcg/L. Prior to initiating chelation therapy, he was referred for a second opinion.

At the time of referral for another opinion, his lab studies demonstrated a ferritin of 1,963 ug/L, TIBC 489, iron 43, and transferrin saturation of 9%. An MRI of the liver demonstrated no evidence of iron overload. Because the H63D mutation may rarely be associated with iron overload when present with other risk factors, screening for porphyria, hemoglobinopathy, autoimmune disease, and viral hepatitis were confirmed to be normal. The association of early onset cataracts in three generations of this family, and the elevated ferritin in the absence of iron overload provided enough support to make the diagnosis of HHCS [6]. We discontinued the phlebotomies. While we considered treating him with oral iron to accelerate his recovery, he preferred to resume his normal diet and replete his iron stores without supplementation.

On his first follow-up visit after a month of freedom from the phlebotomies, he was near his previous level of work and recreational activities. His anemia resolved, and he was released to his primary internist for health maintenance. With his daughter's signed consent, we reviewed her medical record, but there were no ferritin levels documented. The patient's father was on hospice at the time of this diagnosis and we did not pursue lab work to determine his ferritin level. We considered submitting the patient's blood for analysis of the FTL gene, but availability and cost were barriers. Genetic testing is not necessary to make the diagnosis. An isolated elevated ferritin level in the absence of iron overload in 2 or more members of a family demonstrating an autosomal dominant pattern of early onset cataracts is usually sufficient [6].

## 3. Discussion

This case highlights the truth of the much-quoted aphorism that if we listen, the patient will tell us their diagnosis. In this case, the multicontributor past medical history in the electronic medical record noted only that the patient had “cataract surgery” and failed to describe the procedures, one performed in childhood and the other in young adulthood. The family history listed only a cursory list of the usual negatives. The history of cataract surgery in his daughter, which would have surely attracted some further inquiry, was not mentioned. A more detailed past medical history and a thorough family history made the diagnosis of HHCS likely before any images or lab studies were considered.

This presence of the heterozygous H63D mutation in our patient illustrates the importance of interpreting genetic tests with caution when considering hereditary hemochromatosis. Homozygous mutations of the HFE genotypes often do not predict the phenotype of iron overload. The C282Y mutation, a guanine to adenine substitution at nucleotide 845 of the HFE gene which leads to a cysteine to tyrosine substitution at amino acid 282 (C282Y), is variably reported to account for 60–90% of hereditary hemochromatosis when homozygous. Heterozygotes rarely develop iron overload without other risk factors. The H63D mutation, a guanine to cytosine substitution at HFE nucleotide 187 which leads to a histidine to aspartic acid substitution at amino acid 63 (H63D), is much more common than the C282Y mutation and rarely causes iron overload even in homozygotes. Combinations of doubly heterozygous hemochromatosis mutations and heterozygous mutations occurring with other illnesses may result in ferritin elevation with or without iron overload [7]. Pertinent to our patient, an interaction between the H63D mutation and the L-ferritin mutation has not been shown [8].

This case also highlights the lack of awareness of a potential connection between early onset cataracts with elevated ferritin. The prevalence of this disease is considered to be rare, but what was once thought to be a disease located in one or two European countries has now been diagnosed in several areas of the United States and other parts of the world. It is probably better characterized as unrecognized. Many cases are only diagnosed after misdiagnosis of hemochromatosis because of the elevated ferritin and after a significant investment in expensive, painful tests followed by iatrogenic iron deficiency. Ophthalmologists who remove early onset cataracts are often unaware of the HHCS syndrome and do not order iron studies or refer the patient for evaluation. There were missed opportunities in this case. Our patient had two separate cataract surgeries while very young. His elevated ferritin was discovered many years later during an evaluation of abnormal liver functions. His referral to a hematologist was based on the erroneous presumption of iron overload due to the H63D mutation and an elevated ferritin. The patient's daughter, also with two cataract surgeries as a teenager, did not have further evaluation.

## 4. Conclusion

HHCS is an autosomal dominant genetic disorder that causes hyperferritinemia without iron overload and early onset cataracts. Clues to the diagnosis require a detailed medical and surgical history and a thorough family history. It should be considered in any patient with cataracts at a young age, even in the absence of a family history. Anyone found to have an elevated ferritin in the absence of iron overload without another explanation should have an ophthalmology evaluation. Patients should be counseled regarding the autosomal dominant pattern of heredity, the absence of any organ pathology other than eyes, and that the only treatment necessary is cataract surgery.
